# Pathophysiological and behavioral deficits in developing mice following rotational acceleration-deceleration traumatic brain injury

**DOI:** 10.1242/dmm.030387

**Published:** 2018-01-01

**Authors:** Guoxiang Wang, Yi Ping Zhang, Zhongwen Gao, Lisa B. E. Shields, Fang Li, Tianci Chu, Huayi Lv, Thomas Moriarty, Xiao-Ming Xu, Xiaoyu Yang, Christopher B. Shields, Jun Cai

**Affiliations:** 1 Department of Spine Surgery, Orthopedics Hospital affiliated to the Second Bethune Hospital, Jilin University, Changchun 130041, China; 2 Department of Pediatrics, University of Louisville School of Medicine, Louisville, KY 40202, USA; 3 Norton Neuroscience Institute, Norton Healthcare, Louisville, KY 40202, USA; 4 Department of Orthopedics, China-Japan Union Hospital of Jilin University, Changchun 130033, China; 5 Department of Neurological Surgery, China-Japan Friendship Hospital, Beijing 100029, China; 6 Eye Center of the Second Bethune Hospital, Jilin University, Changchun 130041, China; 7 Stark Neurosciences Research Institute, Department of Neurological Surgery, Indiana University School of Medicine, Indianapolis, IN 46202, USA; 8 Department of Neurological Surgery, University of Louisville School of Medicine, Louisville, KY 40202, USA; 9 Department of Anatomical Sciences and Neurobiology, University of Louisville School of Medicine, Louisville, KY 40202, USA

**Keywords:** Abusive head trauma, Shaken baby syndrome, Rotational acceleration-deceleration injury, Ischemia, Hemorrhage, Neuronal degeneration

## Abstract

Abusive head trauma (AHT) is the leading cause of death from trauma in infants and young children. An AHT animal model was developed on 12-day-old mice subjected to 90° head extension-flexion sagittal shaking repeated 30, 60, 80 and 100 times. The mortality and time until return of consciousness were dependent on the number of repeats and severity of the injury. Following 60 episodes of repeated head shakings, the pups demonstrated apnea and/or bradycardia immediately after injury. Acute oxygen desaturation was observed by pulse oximetry during respiratory and cardiac suppression. The cerebral blood perfusion was assessed by laser speckle contrast analysis (LASCA) using a PeriCam PSI system. There was a severe reduction in cerebral blood perfusion immediately after the trauma that did not significantly improve within 24 h. The injured mice began to experience reversible sensorimotor function at 9 days postinjury (dpi), which had completely recovered at 28 dpi. However, cognitive deficits and anxiety-like behavior remained. Subdural/subarachnoid hemorrhage, damage to the brain-blood barrier and parenchymal edema were found in all pups subjected to 60 insults. Proinflammatory response and reactive gliosis were upregulated at 3 dpi. Degenerated neurons were found in the cerebral cortex and olfactory tubercles at 30 dpi. This mouse model of repetitive brain injury by rotational head acceleration-deceleration partially mimics the major pathophysiological and behavioral events that occur in children with AHT. The resultant hypoxia/ischemia suggests a potential mechanism underlying the secondary rotational acceleration-deceleration-induced brain injury in developing mice.

## Abstract (precis)

**Summary:** A neonatal mouse model that mimics abused head trauma in children offers a novel and reliable tool for finding mechanisms underlying the trauma and assessing treatments.

## INTRODUCTION

Abusive head trauma (AHT), also known as shaken baby syndrome, nonaccidental head injury or inflicted traumatic brain injury (TBI), is the leading cause of death from trauma in children aged <2 years, and is a major cause of morbidity in infants and young children (Duhaime et al., 1998). AHT occurs when the head of the child is shaken rotationally in the flexion-extension axis without direct blunt impact. In the United States, shaken baby syndrome is estimated to occur in 14-30 of every 100,000 children during the first year of life (Barlow and Minns, 2000; Herman et al., 2011; Keenan et al., 2003). The true incidence of AHT is probably much higher as many injuries likely go undetected, because minor cases might not be recognized by physicians. Approximately 13-36% of AHT victims die as a result of their injuries (Matschke et al., 2009), and 62%-96% of survivors suffer permanent physical, neurological and mental disabilities (Lind et al., 2013; Matschke et al., 2009). Patients often require long-term care and treatment, which pose a major economic burden to the family and society (Fiske and Hall, 2008).

Greater understanding of AHT relies on longer follow up of patients and use of animal and experimental mechanical models. Large animal models, such as monkeys and lambs, are advantageous owing to their large gyrencephalic brain supported by weak neck muscles, resembling the human infant (Anderson et al., 2014; Finnie et al., 2012, 2010; Gennarelli et al., 1982; Ommaya et al., 1968; Sandoz et al., 2012). Alternatively, pigs and dogs have also been used (Coats et al., 2016; Eucker et al., 2011; Friess et al., 2009, 2011; Naim et al., 2010; Raghupathi and Margulies, 2002; Raghupathi et al., 2004; Serbanescu et al., 2008; Shaver et al., 1996). Rat models can imitate AHT in the infant (Smith et al., 1998; Smith and Hall, 1998). These models partially duplicate the pathology observed in severe AHT seen clinically, including the presence of subdural and subarachnoid hemorrhage, brain swelling, contusion, cerebral laceration, diffuse gliosis, retinal hemorrhage, diffuse axonal injury (DAI) and neurological problems (e.g. cerebral palsy, mental retardation or epilepsy), as well as cognitive and behavioral problems (Beers et al., 2007; Bonnier et al., 1995; Calder et al., 1984; Duhaime et al., 1996; Geddes et al., 2001a,b; Jaspan et al., 1992; Shannon et al., 1998; Vowles et al., 1987; Zimmerman et al., 1979). Murine models can also be utilized in the analysis of the causes of TBI in infants and children and their physiological consequences (Duhaime et al., 1987; Goldsmith and Plunkett, 2004; Pierce and Bertocci, 2008).

During the past decade, genetically modified mice have been used to test novel hypotheses, elucidate pathological mechanisms of brain injuries, and identify putative therapeutic targets. Although the size and shape of the mouse brain and skull and its susceptibility to injury are different from that of humans, studies in mice with different genetic modifications have advanced our knowledge of the pathophysiology and mechanisms of AHT. Unfortunately, modeling of AHT, especially for flexion-extension rotational acceleration-deceleration injury (RADi), has not been developed in mice. Only one mouse model has been reported to mimic AHT (Bonnier et al., 2002, 2004). However, the study has been questioned as to its clinical relevance because the mouse pup was placed on a laboratory horizontally rotating shaker that is not able to produce head acceleration-deceleration motion as occurs in AHT. Here, we introduce a mouse AHT model that rotates the animal head similar to the extension/flexion head motion, reflecting the etiology of AHT. The severity of RADi can be adjusted, and the resultant pathological/functional changes following this injury are evaluated.

## RESULTS

### Survival rate and return of righting reflex

**Fig. 1.**
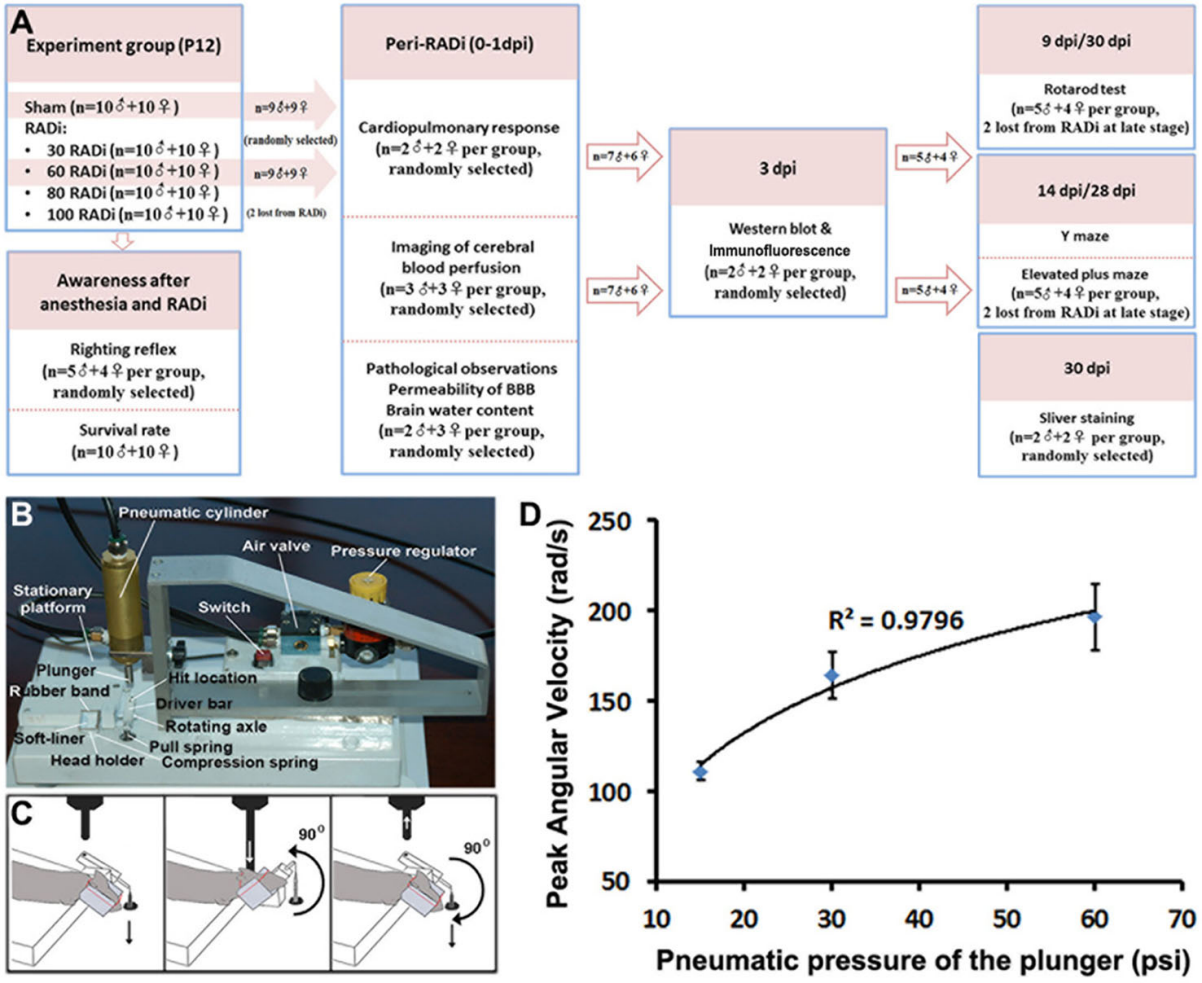
**Study design and RAD brain injury model**. (A) Experimental workflow for rotational acceleration-deceleration TBI in developing mice. (B) Illustration of the major components of the RADi device. (C) Schematic of the RADi procedure. Hyperextension of the neck occurs when the plunger strikes the ‘driver bar’ at the ‘hit location’ site (white down arrow) on activation of the pneumatic cylinder. When the plunger is released (white up arrow), the neck is forced back to the flexed position by the compression spring attached to the anterior part of the rotating axle (black down arrow). Each hyperextension-flexion cycle represents one rotation. (D) Pneumatic pressure of the plunger (psi) versus the peak angular velocities (rad/s) of the head.

No postnatal day (P) 12 pup died as a result of anesthesia or after 30 RADi. However, two, three and four out of a total of 20 pups for each group did not regain spontaneous respiration after 60, 80 and 100 RADi, respectively (Fig. 1A). Recovery of the righting reflex signifying return of consciousness in mouse pups was significantly delayed in an intensity-dependent manner in traumatized pups compared with sham pups (Fig. 1B).

### Cardiopulmonary response

After 60 exposures to RADi of 60 pounds per square inch (psi), all the pups showed central apnea with significantly depressed respiratory rate and tissue oxygenation. Severe oxygen desaturation (<70%) was observed in the pups with not only bradypnea but bradycardia as well. After spontaneous recovery of respiration and the righting reflex, the average respiratory rate temporarily exceeded the control, but the oxygen saturation remained lower than normal (Table 1).

**Table 1.**
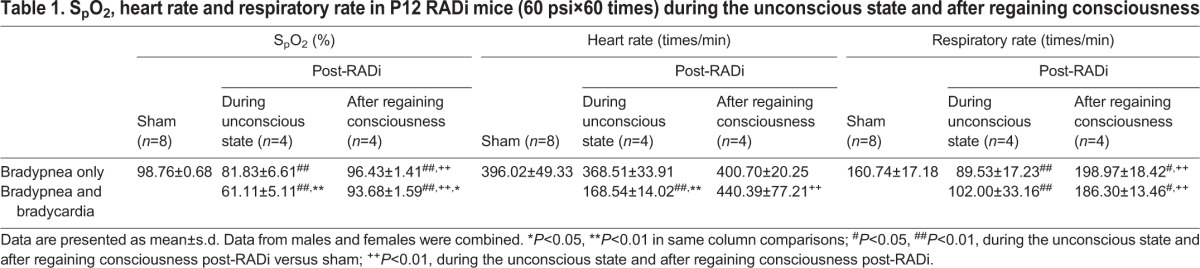
**S_p_O_2_, heart rate and respiratory rate in P12 RADi mice (60 psi×60 times) during the unconscious state and after regaining consciousness**

### Severe reduction in cerebral blood perfusion

**Fig. 2.**
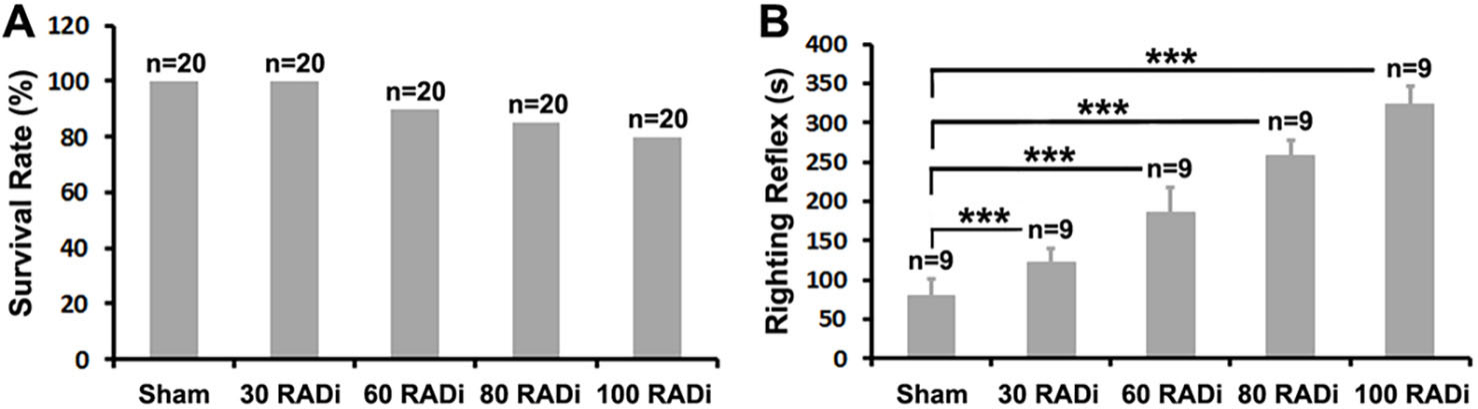
**Survival rate and recovery of the righting reflex.** (A) Survival rate following 60 psi for 30, 60, 80 and 100 RADi (*n*=20 per group). (B) Recovery time of the righting reflex in sham and after 30, 60, 80, and 100 RADi with 60 psi (*n*=9 per group). Data are presented as mean±s.d. and were analyzed by one-way ANOVA followed by Tukey's post hoc test. ***P<0.001.

Baseline measurements were recorded under anesthesia for 5 min prior to the RADi (60 times at 60 psi severity). Cerebral blood perfusion (CBP) measurements were recorded for another 5-min period immediately following the RADi, as well as 4 h and 12 h after RADi. Representative images of CBP are demonstrated (Fig. 2A). Immediately after RADi, a dramatic decrease in blood perfusion occurred throughout the cerebral hemispheres, which improved slightly at 4 h after injury but remained severely depressed at 24 h (Fig. 2B; Table S1). Compared to the baseline, CBP was only 38.9% and 43.3% in the cerebral hemispheres immediately after RADi and 4 h later, respectively. CBP reached 43.9-78.6% of the baseline at 24 h postinjury (Table S2).

### Brain hemorrhage, increased permeability of the brain-blood barrier and water content

**Fig. 3.**
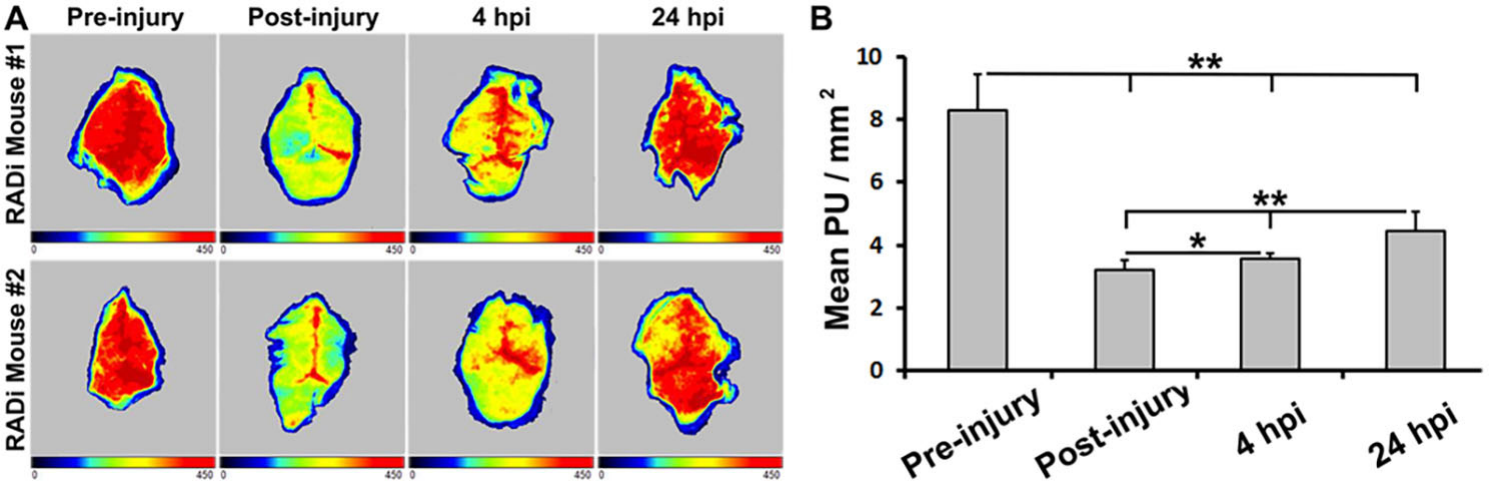
**Cerebral blood perfusion preinjury, immediately after RADi, and 4 h and 24 h following 60 psi×60 RADi.** (A) Representative images of LASCA of blood perfusion in two RADi mouse brains. Baseline and RADi images show areas of yellow-red as high blood perfusion and areas of blue-black as low blood perfusion. (B) Statistical analysis of mean PU per square millimeter. Data are presented as mean±s.d. and were analyzed by one-way with repeated-measures ANOVA followed by Tukey's post hoc test. *P<0.05, **P<0.01; *n*=6.

Fractures and subcutaneous hematomas were not found in the skull, cervical spine and mandible. Bleeding into cervical paraspinal muscles, epi- or subdural spaces rarely occurred. Interstitial edema, vacuolar degeneration and intraspinal hemorrhage were not observed in cervical spinal cords. Brain congestion and intracranial hemorrhage were tightly associated with the frequency of injury (Fig. 3A). Subarachnoid hemorrhage was observed dorsally and ventrally in brains subjected to 60 psi×60 RADi (Fig. 3B, arrowheads). Although deep parenchymal hemorrhages were not identified, Evans Blue was dramatically increased in traumatized brains (712.9±11.6 mg/g dry brain) compared with that in shams (45.0±12.5 mg/g dry brain, *P*<0.05, *n*=5), indicating increased permeability of the brain-blood barrier (BBB). Because increased BBB permeability is associated with acute edema soon after TBI, we measured water content in both sham and traumatized brains. The injured brains had a greater water content than sham brains (5.05±0.24 versus 4.74±0.15 g/g dry brain, *P*<0.05, *n*=5), indicating that acute edema developed when measured at 6 h after injury (Fig. 3C).

### Proinflammatory and glial responses

**Fig. 4.**
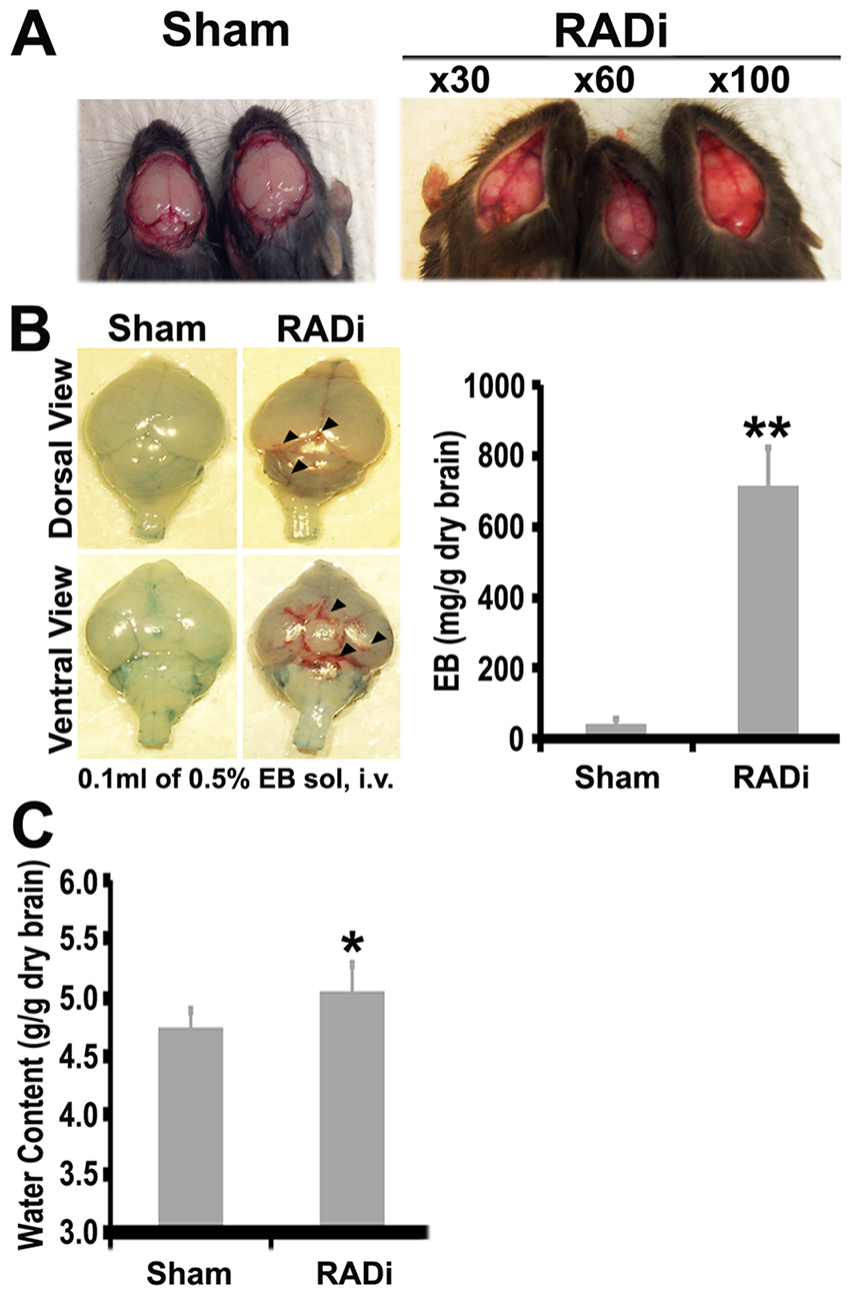
**Hemorrhage and cerebral edema following RADi.** (A) Subdural/subarachnoid hemorrhages of brains of sham mice (left) and mice following different repetitions of RADi at 60 psi (right). (B) Breakdown of BBB integrity owing to increased vascular permeability in sham mice and in mice subjected to 60 psi×60 RADi taken 7 h postinjury. The dramatic retention of Evans Blue in traumatized brains compared with that in sham brains indicates increased permeability of the BBB. (C) Water content in sham and RADi mice. Data are presented as mean±s.d. and were analyzed by unpaired two-tailed Student's *t*-test. *P<0.05, **P<0.01; *n*=5 per group. Arrowheads indicate subarachnoid hemorrhage.

Levels of proinflammatory cytokines, including IL-1β and IL-6, are increased in the cerebrospinal fluid (CSF) after severe TBI in children (Bell et al., 1997; Chiaretti et al., 2005). The cytokine tumor necrosis factor alpha (TNFα) also plays an important role in mediating the inflammatory and immune responses after TBI (Waters et al., 2013). Animal studies indicate that TNFα protects neurons after brain injury (Bruce et al., 1996; Sullivan et al., 1999). In our study, more IL-6 protein, but not TNFα, was detected in injured brains compared with sham brains at 3 dpi. Expression of GFAP and Iba1, specific proteins in astrocytes and microglia, respectively, were significantly elevated (Fig. 4A,B). Furthermore, more Iba1-positive microglia and GFAP-positive astrocytes were found in the ventral pons (Fig. 4C). These observations confirmed an endogenous proinflammatory response and glial activation after RADi.

### Neuronal degeneration in the cerebral cortex

**Fig. 5.**
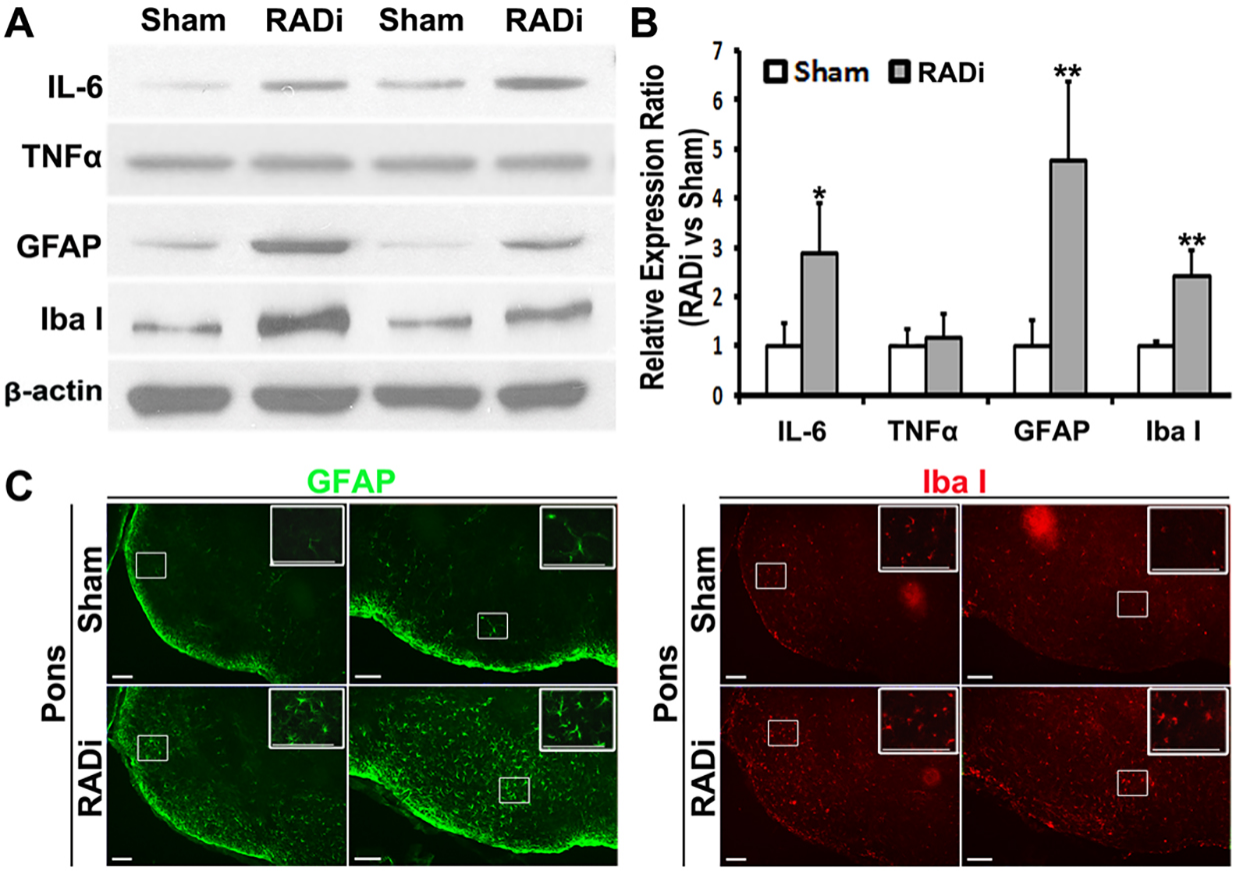
**Proinflammatory and glial activation following RADi in the brain.** (A) Western blots of proinflammatory cytokines IL-6 and TNFα, the glial-specific intermediate filament protein GFAP, and macrophage/microglia-specific protein Iba1 in two sham and RADi mouse brains at 3 dpi following 60 psi×60 RADi. (B) Statistical analysis of western blots. Data are presented as mean±s.d. and were analyzed by unpaired two-tailed Student's *t*-test. *P<0.05, **P<0.01; *n*=4 per group. (C) Photomicrographs of immunostaining showing increased GFAP- and Iba1-positive glia in the pons of sham and RADi brains at 3 dpi. Insets show higher magnifications of the boxed areas. Scale bars: 100 µm.

Silver staining indicates subtle but important degenerative alterations in neurons and/or neural connectivity following trauma. This technique is more sensitive than traditional immunohistochemical methods (e.g. for amyloid precursor protein and/or neurofilament) in the assessment of axonal injury. Neurons undergoing degeneration were demonstrated by dense silver precipitates appearing as black grains (brightfield) in their somata and/or axons. Degenerated neurons were found in the primary motor cortex, primary somatosensory cortex and olfactory tubercles (OT) in the forebrain at 30 dpi following a RADi of 60 psi×60 exposures (Fig. 5). However, axonal degeneration (black staining, axonal bulbs) was rarely observed by either silver staining or immunohistochemical staining with antibodies to neurofilament proteins.

### Neurobehavioral changes

**Fig. 6.**
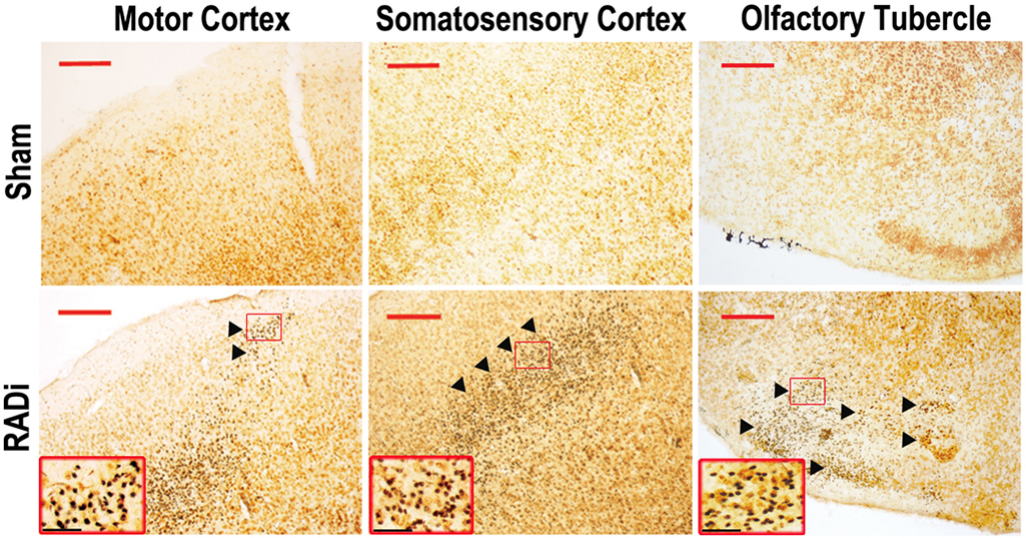
**Neuronal degeneration in RADi mouse cortex at 30 dpi following 60 psi×60 RADi.** Silver staining showed neuronal degeneration (dark cells, arrowheads) in primary motor cortex, primary somatosensory cortex, and olfactory tubercle. Insets show higher magnifications of the boxed areas. Scale bars: 100 μm (red), 25 μm (black).

The proinflammatory response, glial activation and neuronal degeneration in specific brain areas after RADi elicit neurofunctional alterations in mice. Mice subjected to repetitive RADi showed decreased duration on the rotarod than sham littermates at 9 dpi (99.75±21.82 s for sham versus 51.75±30.95 s for RADi; *n*=9, *P*<0.05), which resolved at 28 dpi (139.59±38.45 s for sham versus 147.89±34.28 s for RADi; *n*=9 for sham, *n*=7 for RADi, *P*>0.05) (Fig. 6A). By contrast, the Y-maze score was significantly decreased in RADi at 28 dpi (55.00±6.94% for sham versus 42.92±11.15% for RADi; *n*=9 for sham, *n*=7 for RADi, *P*<0.05), without a significant change in the total number of entries (Fig. 6B). Similarly, there was a robust increase in the time spent in the closed arms of the elevated plus maze (EPM) (200.42±24.44 s for sham versus 239.29±13.91 s for RADi; *n*=9 for sham, *n*=7 for RADi, *P*<0.01) with a decreased interval spent in the open arms (50.72±12.43 s for sham versus 20.96±7.36 s for RADi; *n*=9 for sham, *n*=7 for RADi, *P*<0.01) in injured mice at 28 dpi compared with sham littermates. However, the frequency of entries into the closed or open arms of the EPM was not different between the injured and sham mice. More intriguingly, a significant decrease in the frequency (21.22±3.46 for sham versus 11.22±3.67 for RADi; *n*=9, *P*<0.01) and total duration (22.22±4.75 s for sham versus 10.56±2.73 s for RADi; *n*=9, *P*<0.01) of head dips beyond the borders of the open arms was observed in mice with RADi at 14 dpi, which become progressively worse at 28 dpi (frequency: 20.14±4.67 for sham versus 4.71±1.50 for RADi, *P*<0.01; duration: 21.79±7.24 s for sham versus 5.14±1.60 s for RADi, *P*<0.01; *n*=9 for sham, *n*=7 for RADi) (Fig. 6C).

## DISCUSSION

**Fig. 7.**
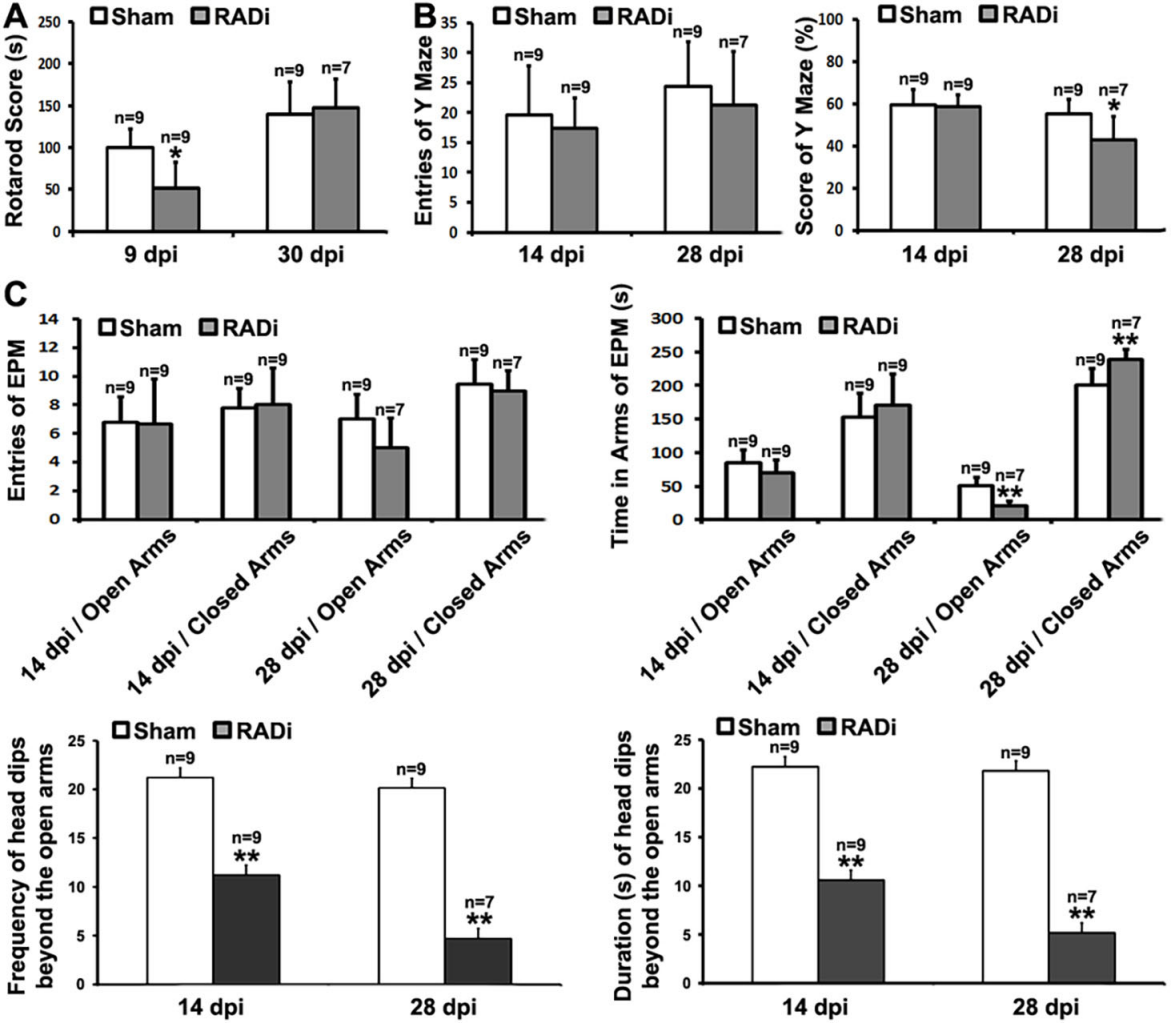
**Neurobehavioral changes in sham and RADi mice.** (A) Rotarod performance at 9 dpi and 30 dpi. Mice that experienced RADi showed much shorter duration on the rotarod than sham mice at 9 dpi but recovered at 30 dpi. (B) Y-maze test at 14 dpi and 28 dpi. Y-maze score but not number of arm entries significantly declined in RADi mice only at 28 dpi. (C) EPM at 14 dpi and 28 dpi. Mice that experienced RADi showed a robust increase in the time spent in the closed arms with a decreased interval spent in the open arms only at 28 dpi. However, a significant decrease in the frequency and total duration of head dips beyond the borders of the open arms was observed in mice with RADi at 14 dpi, which became progressively worse at 28 dpi. Data are presented as mean±s.d. and were analyzed by one-way with repeated-measures ANOVA followed by Tukey's post hoc test. *P<0.05, **P<0.01; *n*=9 for sham, *n*=9 for RADi at 9 dpi and 14 dpi, *n*=7 for RADi at 28 dpi and 30 dpi (two mice were lost for the late stages).

Well-developed neck muscles reflexively protect the head from sudden positional changes. If the magnitude of injury is either too severe or the neck muscles too weak to limit head rotation, brain damage can occur similar to that observed in sports injuries, battlefield blast injuries and AHT (Johnson et al., 2015; Zhang et al., 2014). Rotational acceleration-deceleration (RAD) movement of the head induces brain damage via the inertial force elicited by the unsynchronized motion between the skull and brain. We developed a mouse model that simulates the RAD motion in the anterior-posterior plane and characterized AHT using P12 mice pups (Fig. 7B,C).

Animal models of AHT are essential to test novel hypotheses, pathological mechanisms and therapeutic interventions. The mechanisms contributing to brain injury following AHT, such as excitotoxicity, inflammation and oxidative stress, have not been extensively investigated (Ruppel et al., 2002). Studying these molecular pathways and creating a functional gain or loss mechanism on target molecules using genetically modified mice could provide a powerful approach to increase our understanding of the underlying mechanisms of AHT. Few models of AHT have been described, especially in the mouse, owing to its small and lissencephalic brain in which RADi can produce limited inertial loads and tissue deformation (Margulies and Coats, 2010).

In our preparation, the mouse pup's head rotated in the sagittal plane with no body motion (Fig. 7C). A soft lining was attached to the inner surface of the head holder that prevented the head from sustaining a direct impact. The injury force generated on the brain by the RADi depends on brain mass and acceleration. A dummy model using a 500-g mass mimicking AHT in infants requires an angular acceleration of 1138.54 rad/s^2^ at 4 Hz shaking frequency (Duhaime et al., 1987). Experimental AHT on a 3- to 5-day-old piglet (brain mass 35 g) generates a much higher angular acceleration of 116,701 rad/s^2^ or 34,375 rad/s^2^ during 12 ms or 20 ms, respectively, for one nonimpact axial rotation (Raghupathi and Margulies, 2002; Raghupathi et al., 2004). Creation of an injury model simulating human AHT using smaller animals might require greater acceleration (Coats et al., 2016). Thus, our mouse AHT model generated an applied angular acceleration of 22,616.97±3659.45 rad/s^2^ at 3 Hz RAD frequency. The injury forces generated on a 0.3-g mouse pup brain would be significantly smaller than that generated in humans and piglets. However, the severity of AHT depends not only on the rotational velocity and brain mass but also on the number of injury repetitions (Coats et al., 2016; Unterharnscheidt and Higgins, 1969). Multiple RADi exposures have a cumulative effect that contributes to the severity of brain damage (Anderson et al., 2014; Coats et al., 2016; Finnie et al., 2012, 2010; Friess et al., 2009, 2011). Thus, the severity of brain damage and pathophysiological manifestations depend on the velocity and number of injury repeats in mice. The greater the severity of RADi and the larger the number of injury repetitions, the higher will be the mortality rate, and the longer will be the duration of postinjury unconsciousness (Fig. 1 and Fig. 7D), which might induce more severe brain damage.

Children who have sustained RAD brain injury might demonstrate poor feeding, vomiting, cardiorespiratory difficulties (apnea and/or bradycardia), subdural/subarachnoid/retinal hemorrhage, gliosis, cerebral contusions, diffuse axonal injury (DAI), and long-term neurological and behavioral problems. Specific patterns of retinal hemorrhage are used to screen victims sustaining AHT (Maguire et al., 2009; Minns et al., 2012). Approximately 57-77% of children with AHT experienced at least one episode of significant apnea (Geddes et al., 2001a; Johnson et al., 1995; Kemp et al., 2003; Maguire et al., 2009). Consistent with clinical observations, mice subjected to RADi experienced a 40% decline in respiratory function including several apneic episodes during the first couple of minutes after injury. Some mice demonstrated significant bradycardia, with a decrease in pulse of >50% from baseline. Episodes of apnea, bradypnea and bradycardia contributed to the development of moderate-to-severe hypoxemia (Table 1). Alterations in respiratory function and oxyhemoglobin desaturation were not completely correctable, which might be attributed to damage of brainstem respiratory centers. However, the precise underlying mechanism(s) of this phenomenon remain unclear.

RADi leads to altered cardiopulmonary function and deranged intracranial dynamics. Following RADi in our study, CBP was dramatically reduced in the entire cerebral hemisphere within the first few hours and had not completely recovered to baseline by 24 h (Fig. 2). The widespread reduction of CBP indicates secondary impairment of CBP regulation, which differs from the regional decline of CBP following focal cerebral contusion (Fig. S2). It suggests a special responsive mechanism of cerebrovascular regulation after RADi. Significant CBP reduction was observed in the piglet following sagittal, but not coronal or horizontal, nonimpact head rotations (Coats et al., 2016; Eucker et al., 2011). The severe reduction of CBP is presumably caused by immediate damage to the cardiopulmonary response (Table 1) (Friess et al., 2011), brainstem impairment and/or cerebral vasospasm (Clevenger et al., 2015; Friess et al., 2011; Izzy and Muehlschlegel, 2013). Cerebral oxygenation relies on CBP, arterial content of oxygen and cerebral oxygen consumption. A severe reduction of CBP suggests the presence of secondary brain damage caused by ischemia/hypoxia.

During sudden sagittal rotational movement of the head, acceleration-deceleration motion can induce enough shear force to tear superficial vessels. Subdural hematomas and subarachnoid hemorrhage were observed over the dorsal and ventral brain surfaces, including the rostral and caudal cerebrum, colliculus, cerebellum, optic chiasm, median eminence and rostroventral medulla, in injured mice (Fig. 3A,B). Similar findings have been observed following AHT in piglets (Coats et al., 2016; Raghupathi and Margulies, 2002). Shear stress and hypoxia can alter tight junction proteins of the endothelium (Tarbell, 2010; Yamagata et al., 2004), causing cerebral edema through breakdown of the BBB and fluid extravasation (Fig. 3B,C). Retinal hemorrhage was reported in ≤85% of children with AHT (Levin, 2010; Maguire et al., 2009; Morad et al., 2010), with a different pattern of hemorrhage observed following AHT from that seen in non-AHT (Minns et al., 2012; Yu et al., 2012). Retinal hemorrhage was uncommon in mice subjected to RADi (one in nine cases, Fig. S1). Retinal hemorrhages are rarely produced in rodents (Bonnier et al., 2004; Serbanescu et al., 2008) but occur more frequently in larger animals (Ommaya et al., 1968). This difference might be caused by greater shear stresses that are generated in the larger human brain. In addition, repeated head rotations at low velocities do not induce ocular injury in the piglet (Coats et al., 2016).

Apnea, bradypnea/bradycardia and CBP reduction following AHT enhance the damaging effect of hypoxa/ischemia on the traumatized brain (Eucker et al., 2011; Kemp et al., 2003; Naim et al., 2010). Inflammatory responses and diffuse gliosis were observed in patients following AHT and in other animal models of head injury (Bonnier et al., 2002; Calder et al., 1984). Proinflammatory changes and glial activation were identified in our mice at 3 dpi (Fig. 4). DAI, characterized by axonal swelling and varicosities along the axons as well as the presence of large terminal bulbs, is frequently seen following TBI (Siedler et al., 2014), and is observed in >70% of patients seen clinically (Skandsen et al., 2010). DAI in children with AHT has been reported in some studies (Calder et al., 1984; Reichard et al., 2003; Shannon et al., 1998; Vowles et al., 1987), but not in others (Dolinak and Reichard, 2006; Geddes et al., 2001b; Geddes and Whitwell, 2004; Maguire et al., 2009). In a monkey study, DAI was reported following head motion in the coronal plane but not after head motion in the sagittal plane (Gennarelli et al., 1982). However, widespread swollen and disconnected axons (bulbs) as well as β-APP-positive degenerated neurons were observed in neonatal piglets (Coats et al., 2016; Eucker et al., 2011; Raghupathi and Margulies, 2002; Raghupathi et al., 2004) and lambs (Finnie et al., 2010; Friess et al., 2011) following RADi in the sagittal plane. Although DAI was rarely detected in mice 1 month after RADi [anti-β-APP and anti-NF-H (NEFH) immunohistochemical staining data not shown] in the present study, progressive neuronal degeneration occurred in mouse brains subjected to RADi at 30 dpi (Fig. 5). Neuronal degeneration was seen in the motor/somatosensory cortex and the OT. Whether delayed DAI occurs in mice after RADi needs to be studied by electron microscopic or array-tomography-based approaches. DAI might be created in mouse brains after repeat rotational-acceleration insults over many days because the density and distribution of injured axons in immature brains are associated with a graded response to the severity of injury (Raghupathi et al., 2004). The degenerated neurons are distributed from layer II to layer V in the motor/somatosensory cortex. These laminae are the main regions of inter- and intrahemispheric corticocortical afferents (layers II-IV), thalamocortical afferents (layer IV), principal corticocortical efferents (layer III), efferents to the basal ganglia and corticospinal tract (layer V), which are involved in cognition, emotion and voluntary movements (DeFelipe, 2011). The OT is a multisensory processing center in the basal forebrain, which is interconnected with numerous other brain regions to form a critical interface between processing of sensory information and subsequent behavioral responses (Wesson and Wilson, 2011). Therefore, neuronal degeneration in those regions probably contributes to changes of neurological and behavioral function that manifest in a delayed fashion (Fig. 6). It has been reported that following TBI in children, some neurobehavioral deficits do not emerge until adulthood (Semple et al., 2012). Evaluation of behavioral outcome at 3-6 months might provide additional value using this model. In order to identify the interdependency of vulnerable neurons and malfunction, the cell types/subpopulations of those degenerated neurons (Zeisel et al., 2015) and more analyses of long-term behavioral changes (Guida et al., 2017; Milman et al., 2005), including nociceptive response, depression-like activity and sociability, are worthy of further investigation. The serum and/or CSF concentrations of neuron-specific enolase (NSE; ENO2), S100B, myelin-basic protein (MBP), interleukin 6 (IL-6), vascular cell adhesion protein (VCAM1), and cortisol levels (Berger et al., 2005, 2006, 2002, 2009; Heather et al., 2012) were detected in children with AHT. Further study using animal models such as ours could allow screening for biomarkers that will be more specific at measuring the severity and predicting the prognosis of AHT.

Animal models of RADi of the brain might offer a reliable preclinical/translational tool to identify biomarkers and assess therapeutic interventions for children with AHT. In this study, AHT was produced in neonatal mice. Under the applied severity of RADi (60 psi) and number of injury repetitions (60 insults), many pathophysiological and functional changes were noted in the mice, including the presence of subdural and subarachnoid hemorrhage (Fig. 3A,B), brain swelling (Fig. 3C), diffuse gliosis (Fig. 4), retinal hemorrhage (Fig. S1), neuronal degeneration (Fig. 5), and cognitive and behavioral problems (Fig. 6), that are comparable to those that occur in children with severe AHT as well as large animal models. More detailed pathophysiological study in different brain regions (e.g. hippocampus, basal forebrain and brainstem) correlating with behavioral deficits, long-term sequelae, the underlying mechanism(s) and potential interventions will be investigated in mice after RADi. In addition, a biofedelic model to mimic the neonatal mouse will be developed to further validate this model.

## MATERIALS AND METHODS

### Animals

Male and female C57BL/6 mice (Jackson Laboratories, Bar Harbor, ME) were bred onsite for this study. P12 C57/BL6 mouse pups were culled by body weight (6.0±0.4 g) from each litter and randomly divided into five groups for assessment of righting reflex and mortality after injury: a sham group as control and groups subjected to 30, 60, 80 and 100 RAD insults (*n*=20 for each group, 10 males and 10 females). Then, the sham and 60 RADi groups were selected for pathophysiological, imaging, morphological, behavioral and gene expression studies. The experimental groups, animal numbers and procedures are described in the flow chart (Fig. 7A). All protocols were approved by the University of Louisville Research Resources Center, an American Association for Laboratory Animal Care-approved facility, and performed in accordance with the guidelines of the Animal Care and Use Committee of the University of Louisville School of Medicine and NIH requirements for the care and use of laboratory animals. After RADi and posttraumatic evaluation of recovery of consciousness and cardiopulmonary function, buprenorphine (2.0 mg/kg body weight, Sigma-Aldrich, St. Louis, MO) was administered subcutaneously to control pain in both sham and injured pups before being returned to their lactating dam and during the following 3 days.

### RAD brain injury model

P12 postnatal mice pups were chosen because myelination, axon development and synapse formation in such brain were roughly equivalent to that in the brain of 1-year-old children (Romijn et al., 1991; Semple et al., 2013). Thus, P12 mice mimic the age of typical AHT patients (Curristin et al., 2002; Fagel et al., 2006; Li et al., 2009). The mouse pup was anesthetized using 3% isoflurane in 100% oxygen for 150 s in an anesthesia box. The pup was removed from the box after being anesthetized and placed on a stationary platform in the prone position. The pup's head was placed and fixed by an elastic band in the rotatable head holder at a flexed starting position, and its body was immobilized on the platform at the thoracic level. The head was hyperextended along the sagittal axis when the head holder was activated by compressed air via a pneumatic cylinder, causing the plunger to strike the driver bar. After each extension movement, the neck was flexed by a spring-loaded mechanism (Fig. 7B,C). Each RADi represented one cycle of extension-flexion in a sagittal rotation. The velocity of rotational acceleration and angular limits were adjusted by altering the pneumatic pressure (psi) and stroke distance of the plunger. A 25-mm plunger stroke generated a rotational angle of 90° (Fig. 7C). The frequency of rotation was set at 3 Hz to simulate previous reports (3-5 Hz) using an anthropometric dummy (Duhaime et al., 1987; Goldsmith and Plunkett, 2004). The repetitive RADi generated an AHT model. The maximum linear velocity (m/s) of the plunger that induced head extension acceleration from its flexed starting position was measured by the distance change in unit time using a laser distance sensor (OADM 12 U6430; Baumer, Southington, CT). The head rotating time was calculated based on the maximum linear velocity and stroke distance of the plunger. The maximum angular velocity (radian per second, rad/s) and maximum angular acceleration of head rotation (rad/s^2^) were derived from the basic formula of angular velocity and angular acceleration (Halliday et al., 2013) as follows:

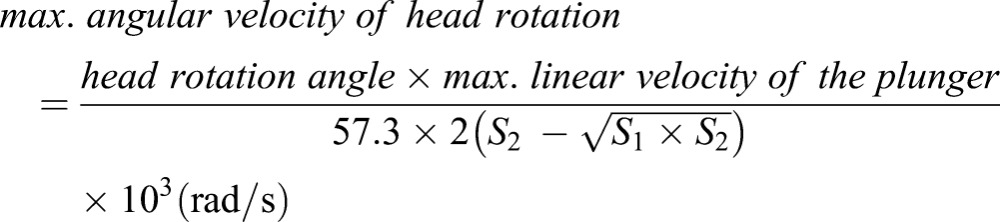


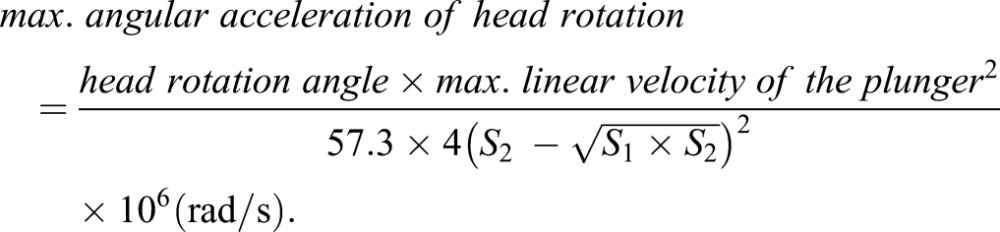


Head rotation angle=90°; S_1_, distance that the plunger moves till reaching the driver bar=7 mm; S_2_, full distance that the plunger moves=25 mm.

The maximum speed of head rotation was dependent on the pneumatic pressure in a logarithmic manner (Fig. 7D). The maximum angular velocity and acceleration of the head rotation using the 60 psi pneumatic pressure were 196.30±18.39 rad/s and 22,616.97±3659.45 rad/s^2^, respectively.

### Righting reflex

Neonatal mice, as early as 2 days after birth, normally assume a prone position within 5 s after being placed on their backs (righting reflex). The sex of the mouse does not affect the righting reflex response or its latency (Dierssen et al., 2002; Fox, 1965; Le Roy et al., 2001). The latency of the righting reflex was used to indicate the capability to regain consciousness or proprioception of the animals after anesthesia and injury in this study. An alteration in the state of consciousness is a key characteristic of a cerebral concussion. Each pup was placed on its back after the injury, and the time taken for the pup to attain the prone position with four paws on the ground was recorded as the recovery latency. To exclude the anesthetic effect of isoflurane, the righting reflex was also tested on sham animals subjected to anesthesia without injury.

### Cardiopulmonary function and pulse oxyhemoglobin saturation

The procedure was modified from our previous report (Cai et al., 2011). After induction of anesthesia with isoflurane, cardiopulmonary function and pulse oxyhemoglobin levels were measured in pups by clipping a sensor to the right thigh. These functions were recorded in P12 sham mice and in mice after the RADi (unconscious) until the mice resumed normal respiration and regained consciousness. Respiratory rate, heart rate and arterial oxygen saturation (S_p_O_2_) were monitored using a MouseOxTM Oximeter (STARR Life Sciences, Oakmont, PA). Central apnea was defined as a lack of breathing effort longer than 1 s (Hodges et al., 2009). Analog data were continuously digitized by a computer interfaced using the WinDaq data acquisition system provided by the manufacturer. Data were collected immediately after the RADi and after regaining spontaneous respiration/consciousness following RADi, and analyzed with WinDaq Waveform Browser software (DATAQ Instruments, Akron, OH) during periods without error signals.

### Pathology

The sham and injured pups sustained repeated RAD insults and were perfused intracardially 7 h later with 5 ml cold 0.1 M PBS. The skull, cervical spines and mandible were exposed. Evidence for fracture and hematoma were examined macroscopically in those regions. After performing a craniotomy and laminotomy, epidural/subdural hematoma and subarachnoid hemorrhage were evaluated. Hematoxylin-Eosin (HE) staining was also performed on sections of cervical spinal cords.

### Permeability of the BBB

The BBB assay measures changes in vascular permeability. The procedure was previously described with some modifications (Wang et al., 2016a). Briefly, 0.1 ml of 0.5% Evans Blue (EB, Sigma-Aldrich) in saline was slowly injected into the jugular vein 6 h postinjury. One hour later, the pup was perfused intracardially with 5 ml cold 0.1 M PBS. The brain was dissected, washed, weighed, dried for 48 h and weighed again. EB extravasation was evaluated by formamide incubation (1 ml) for 24 h. The amount of EB in tissue extracts was measured by absorbance at 610 nm as an index of increased capillary permeability. Data were collected from five sham and five RADi mice and shown as the amount of EB (mg) per gram of dry brain tissue. Blinded experiments were performed for data acquisition and analysis.

### Brain water content

Brain edema was determined by measurement of brain water content (Keep et al., 2012). The entire fresh brain was weighed as wet weight immediately after its removal and then placed in an oven for 48 h to obtain dry weight. The brain water content was expressed as (wet weight−dry weight)/dry weight (g/g dry weight). Experiments were performed with blinded samples.

### Fluorescein retinal angiography

Fluorescein angiography was performed in P12 RADi (60 RAD insults) and sham mice. First, 100 μl of 10% fluorescein sodium solution (Hub Pharmaceuticals, Rancho Cucamonga, CA) was intraperitoneally injected into deeply anesthetized sham and injured mice 7 h after injury. Ninety seconds later, the eyes were removed and rinsed in PBS. The cornea, lens and neurosensory retina were carefully removed from the eye. Four radial cuts were made from the edge of the cornea to the equator. The retinal pigment epithelium-choroid-sclera complex was flat mounted in 50% glycerol containing PBS with the sclera against the glass slide. Images of blood vessels and hemorrhage were recorded using an epifluorescence microscope (Nikon Eclipse E800, Nikon Instruments, Melville, NY). Experiments were performed with blinded samples.

### Real-time imaging of CBP

CBP was assessed using a blood perfusion imager (PeriCam PSI System, Perimed AB, Stockholm, Sweden) based on laser speckle contrast analysis (LASCA) technology. Briefly, the P12 mouse pup was transferred into an anesthesia box for 150 s and exposed to 3% isoflurane mixed with 100% oxygen. The mouse was placed in the prone position on a heated pad with a rectal temperature probe, and anesthesia was maintained with a continuous flow of isoflurane. The skull was exposed by initially creating a midline skin incision. The through-skull laser detected movement of red blood cells that created a speckle contrast. Measurement of contrast fluctuations provided information about CBP. After the mouse body temperature reached 37±0.5°C, the dynamic and spatial distribution of blood perfusion was recorded for 5 min in real time by PSI scanning. Images and data were collected from six pups before RADi (baseline), immediately following RADi, as well as at 4 h and 12 h after RADi. Cortical blood perfusion was expressed in arbitrary units (perfusion units, PU).

### Western blots

The entire fresh brain was removed for protein preparation from either four sham or four RADi mice at 3 dpi. Protein samples were prepared in CelLytic™ MT Cell Lysis Reagent (Sigma-Aldrich) plus Complete Protease Inhibitors (Roche, Indianapolis, IN) at 4°C. Western blots were performed as described previously (Cai et al., 2012). Equivalent total protein amounts were loaded onto 7% or 10% polyacrylamide gels (Bio-Rad, Hercules, CA) and then transferred to Protan BA83 Nitrocellulose Membranes (Midwest Scientific, Valley Park, MO). Blots were probed and recognized with the following primary and secondary antibodies: mouse anti-GFAP (1:4000; 3670, Cell Signaling, Danvers, MA), rabbit anti-IL6 (1:3000; AB1423, Millipore, Billerica, MA), goat anti-TNFα (1:100; sc-1350, Santa Cruz Biotechnology, Dallas, TX), rabbit anti-Iba1 (1:1000; 019-19741, Wako, Richmond, VA), mouse anti-β-actin (1:5000; A5316, Clone AC-74, Sigma-Aldrich), and horseradish peroxidase-linked goat-anti-mouse (1:3000; sc-2005), goat-anti-rabbit (1:3000; sc-2006), or donkey-anti-goat (1:3000; sc-2020) (all Santa Cruz Biotechnology). Signals were developed by using chemiluminescence with ECL western blotting detection reagent (Pierce, Grand Island, NY) that was then exposed to film. The optical density (OD) of bands on western blots was measured using ImageJ software (NIH, Baltimore, MD). The ODs for specific proteins were normalized over the ODs for β-actin, and these values were expressed as the ratio relative to the sham control. The experiments were performed with blinded samples.

### Immunofluorescence

Single immunofluorescence on cryostat spinal sections was performed as described previously (Wang et al., 2016b). Images were obtained using an epifluorescence microscope (Nikon Eclipse E800). Antibodies were commercially available: mouse anti-GFAP (1:1000; 3670, Cell Signaling Technology, Danvers, MA) and rabbit anti-Iba1 (1:500; 019-19741, Wako, Richmond, VA). The experiments were performed with blinded samples.

### Silver staining

The entire brain was dissected from either four sham or four RADi mice at 30 dpi after perfusion with 4% paraformaldehyde. Coronal sections of 40-μm thickness were cut on a cryostat. Staining was performed on free-floating brain sections using an FD NeuroSilver™ Kit II (FD Neurotechnologies, Columbia, MD) following an amino-cupric silver histochemical technique (de Olmos et al., 1994; Wang et al., 2016a). The FD NeuroSilver™ Kit II is designed to selectively enhance the staining of degenerating neurons and/or axons while suppressing or eliminating the staining of normal ones. Degenerated neurons were indicated by dense silver precipitates that appeared as black grains (brightfield) in their somata and/or axons. Micrographic images were recorded using a Nikon 800 microscope. Experiments were performed with blinded samples.

### Behavioral assessment

The same nine mice (five males and four females) in both the RADi and sham groups were used for all behavioral tests. Data acquisition and analysis were performed via blinded controls.

#### Rotarod performance

The test was used to assess locomotor function and coordination. A 2-day training/test regimen was adopted for mice on the rotarod (Ugo Basile 7650 accelerating RotaRod, Varese, Italy) with an accelerating speed from 2 rpm to 40 rpm in 600 s as described previously (Wang et al., 2016b). Each trial was recorded from the time the rotarod began turning to the point when the mouse fell off and three trials were conducted. The test was conducted at 9 dpi (weanling time) and 30 dpi, because sensorimotor reflexes and motor skills normally appear with a definite timing during the first 3 postnatal weeks (de Souza et al., 2004), and the cortex develops continuously with changes during the first 3 months (Hammelrath et al., 2016). The average duration of three trials represented the rotarod score.

#### Y-maze spontaneous alteration test

This test was used to measure the rodents' innate tendency to explore a novel environment and spatial working memory (Dellu et al., 1992; Sarter et al., 1988). The apparatus for Y-maze testing is made of three opaque plastic arms (labeled as A, B and C; dimensions: 1.375 inches×7.875 inches) with 6-inch high walls at 120° to each other. The mouse was introduced onto the center of the maze and allowed to explore the three arms freely for 8 min. The number and sequence of entries into each arm was recorded. A complete entry was considered to have occurred when all four limbs entered an arm of the Y-maze. Entry into three different arms in succession (e.g. ABC, BCA, CBA or CAB arms) was defined as one alternation. In the weanling mice, spatial learning and memory can be formed and detected as early as P24 (Barnhart et al., 2015); thus, Y-maze scores were calculated at 14 dpi and 28 dpi by the percentage of alternations in the total number of entries minus 2.

#### EPM

This maze consists of opaque Plexiglas with opposite facing two open arms (dimensions: 14 inches×2 inches) and two enclosed arms (dimensions: 14 inches×2 inches with 6-inch high walls) connected by a central open platform (dimensions: 2 inches×2 inches). The whole maze was raised at least 30 inches off the floor. The mouse was placed at the center of the maze with the head facing an open arm and allowed to explore for 5 min. The number of entries, time spent in each arm, and frequency and total duration of head dips beyond the borders of the open arms were recorded at 14 dpi and 28 dpi. An increase of the time spent and entry frequency in the closed arms, as well as a decrease of frequency and duration of head dips relative to the control animal, were considered indicators of anxiety-related behavior (Walf and Frye, 2007).

### Statistical analysis

Data are presented as mean±s.d. Comparisons between sham and RADi groups were conducted using one-way or repeated-measures analyses of variance (ANOVA) and unpaired two-tailed Student's *t*-tests, as appropriate, followed by the post hoc Tukey's test. The analysis was initially performed based on sex difference for the behavioral test. Because the main effects of sex were not significant, data from males and females were combined. The significance level was *P*<0.05.

## Supplementary Material

Supplementary information
