# Supplementary material for: Pathophysiological and behavioral deficits in developing mice following rotational acceleration-deceleration traumatic brain injury
Source: Dis Model Mech. 2018 Jan 1;11(1):dmm030387. doi: 10.1242/dmm.030387 (PMC5818073; doi:10.1242/dmm.030387)
Supplement: Supplementary information [file dmm-11-030387-s1.pdf]

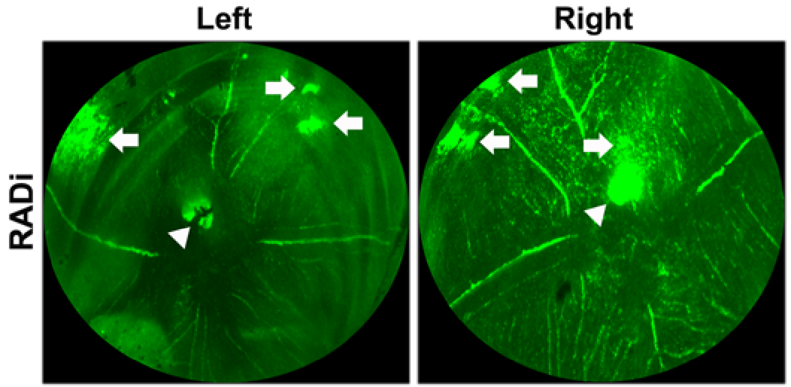

Supplemental Figure 1. Representative images of retinal hemorrhage following 60 psi × 60 RADi. Retinal hemorrhage was reported in up to 85% of children with AHT and more frequently in larger animals but rarely produced in rodents. In this RADi mouse model, retinal hemorrhage occurred uncommonly (1 out of 9 cases). Arrowheads point to the optic discs and arrows indicate areas of hemorrhage.

Disease Models & Mechanisms • Supplementary information

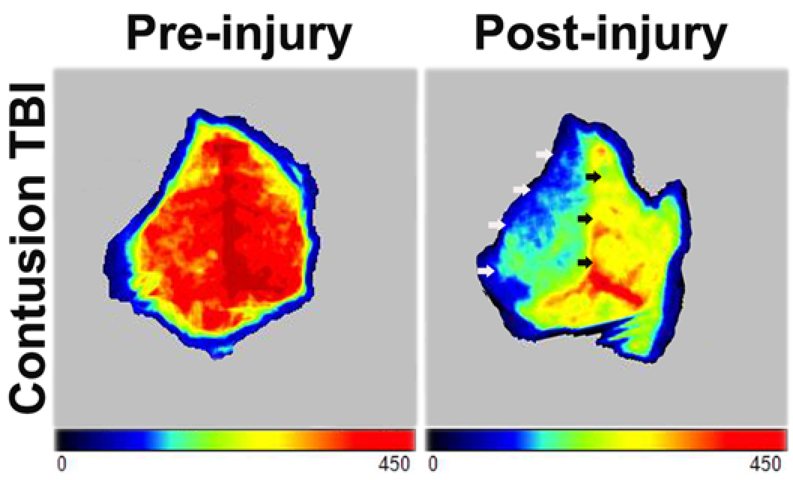

Supplemental Figure 2. Cerebral blood perfusion (CBP) immediately following a focal cerebral contusion injury shown on the left side of the image of a P12 mouse. Reduction of perfusion was greater on the injured side (white arrows) than on the uninjured side (black arrows), not as the widespread reduction of CBP that occurred after RADi. TBI: traumatic brain injury.

Disease Models & Mechanisms • Supplementary information

Table S1. S_pO_2, HR and RR in P12 RADi mice (60 psi x 60 times) during the unconscious state and after regaining consciousness

	S_pO_2 (%)			HR (times/min)			RR (times/min)		
	Sham (n=8)	Post-RADi		Sham (n=8)	Post-RADi		Sham (n=8)	Post-RADi	
		During unconscious state (n=4)	After regaining conscious-ness (n=4)		During unconscious state (n=4)	After regaining conscious-ness (n=4)		During unconscious state (n=4)	After regaining conscious-ness (n=4)
Bradypnea only	98.76± 0.68	81.83± $6.61^{\#\#}$	96.43± $1.41^{\#\#,++}$	396.02± 49.33	368.51± 33.91	400.70± 20.25	160.74± 17.18	89.53± $17.23^{\#\#}$	198.97± $18.42^{\#,++}$
Bradypnea and Bradycardia		61.11± $5.11^{\#\#},^{**}$	93.68± $1.59^{\#\#,++,*}$		168.54± $14.02^{\#\#,**}$	440.39± 77.21^{++}		102.00± $33.16^{\#\#}$	186.30± $13.46^{\#,++}$

RADi: rotational acceleration-deceleration injury; psi: pound per square inch; P12: postnatal day 12; S_pO_2: pulse oxygen saturation; HR: heart rate; RR: respiratory rate. Data were shown as mean±SD. Data from males and females were combined. * represents p<0.05 and ** represents p<0.01 in the same column comparison; $^{\#}$ Represents p<0.05 and $^{\#\#}$ represents p<0.01, during the unconscious state and after regaining consciousness post-RADi vs sham; $^{++}$ represents p<0.01, during the unconscious state and after regaining consciousness post-RADi

Supplemental Table 2. Percent of cerebral blood perfusion compared to the baseline in P12 RADi mice

RADi Mouse #	Pre-injury	Post-injury	4 hpi	24 hpi
1	100	39.0	41.7	43.9
2	100	38.3	42.4	46.6
3	100	39.6	40.5	42.1
4	100	44.7	51.3	78.6
5	100	39.9	45.9	62.6
6	100	32.0	37.7	56.9
Mean ± SD		38.9±4.1	43.3±4.8	55.1±14.0

RADi: rotational acceleration-deceleration injury (60 psi x 60 times); psi: pound per square inch; P12: post-natal day 12; hpi: hour(s) post-injury; Data were shown as percent of cerebral blood perfusion compared to the baseline (pre-injury).
